# Associations of dietary patterns with obesity and weight change for adults aged 18–65 years: Evidence from the China Health and Nutrition Survey (CHNS)

**DOI:** 10.1371/journal.pone.0279625

**Published:** 2023-01-25

**Authors:** Yang Chen, Minji Kang, Hyojin Kim, Wanghong Xu, Jung Eun Lee

**Affiliations:** 1 Department of Food and Nutrition, College of Human Ecology, Seoul National University, Seoul, Korea; 2 BK21 FOUR Education and Research Team for Sustainable Food and Nutrition, Department of Food and Nutrition, College of Human Ecology, Seoul National University, Seoul, Korea; 3 Department of Epidemiology, Fudan University School of Public Health, Shanghai, People’s Republic of China; 4 Research Institute of Human Ecology, Seoul National University, Seoul, Korea; West China Second University Hospital, Sichuan University, CHINA

## Abstract

**Background:**

Obesity has become a significant public health problem within China. By observing dietary patterns, an individual’s actual dietary habit of nutrients or foods in combination can be identified. We aimed to examine dietary patterns in relation to the risk of obesity (body mass index (BMI) ≥ 25 kg/m^2^) and weight change (average weight change per five years) within a Chinese cohort.

**Methods:**

We analyzed the data from 6,677 adults aged 18–65 years in the China Health and Nutrition Survey 1997–2015. Westernized, traditional Chinese, and high-starch plant-based dietary patterns were identified by principal components analysis. We estimated relative risks (RRs) and least-squares means (LS-means) with 95% confidence intervals (CIs) using the Cox proportional hazards models and the generalized linear models, respectively.

**Results:**

High adherence to the Westernized dietary pattern was associated with increased risks of obesity and weight gain. Comparing top with bottom quintiles, RR (95% CI) for obesity (BMI ≥ 25 kg/m^2^) was 1.57 (1.26–1.95; P for trend < 0.001). LS-means of weight change (kg/5 years) were 1.73 (0.98–2.47) and 1.13 (0.39–1.87; P for trend = 0.036) in the top and bottom quintiles, respectively. Increased weight gain among those following the Westernized dietary pattern was stronger in the Southern region than the Northern region. There was a slight hint of an inverse association between the traditional Chinese dietary pattern and obesity. We did not find any significant association for the high-starch plant-based dietary pattern.

**Conclusions:**

The Westernized dietary pattern increased the risk of obesity among Chinese adults. Weight gain associated with Western dietary pattern was more pronounced in the Southern region than in the Northern region. Our study will provide helpful data in developing dietary guidelines for the prevention of obesity specific to different regions in China.

## Introduction

The prevalence of obesity has steadily increased worldwide over the past several decades. According to the 2016 World Health Organization (WHO) report, the prevalence of overweight (body mass index (BMI) ≥ 25 kg/m^2^) or obesity (BMI ≥ 30 kg/m^2^) among adults aged 18 years and above was 39% and 13%, respectively [1]. The epidemic of obesity is observed throughout all countries, not limited to their income status [2]. Accompanied by rapid urbanization and motorization, the prevalence of obesity (BMI ≥ 27.5 kg/m^2^) in China has dramatically increased from 4.2% in 1993 to 15.7% in 2015 [3]. Obesity is an important risk factor for various chronic diseases, including type 2 diabetes [4], cardiovascular disease [5], and several types of cancer [6]. It has been suggested that the cause of obesity is multifactorial, including genetic, environmental, and lifestyle factors [7]. The modification of diet in lifestyle may play a role in the prevention of obesity [8].

Nutrients and foods are consumed in various combination, resulting in high collinearity among foods and nutrients. Therefore, dietary pattern analysis may provide meaningful insights into the relationship between diet and the risk of diseases [9]. Several studies have examined the relationship between dietary patterns and obesity. A recent meta-analysis including 17 observational studies reported a lower risk of overweight or obesity with a healthy or prudent diet pattern, but a higher risk following an unhealthy or Western pattern [10]. Two of the 17 studies from the meta-analysis as well as four separate studies were analyzed the Chinese population using a cross-sectional design to estimate obesity prevalence among young women aged 18–44 years [11], adolescents aged 10–12 years [12], and adult males and females [13–16].

Considering the limited evidence from long-term cohort studies, we aimed to examine prospectively whether dietary patterns were associated with obesity and weight change in the China Health and Nutrition Survey (CHNS). The results of the CHNS study can provide a new approach to prevent the growing epidemic of obesity in China.

## Materials and methods

### Study design and population

The CHNS, an ongoing prospective household-based cohort study, was initiated in 1989. Details of the CHNS are described elsewhere [17]. The CHNS was designed to explore the effect of social, economic, and demographic changes on the health status of the Chinese population. Follow-up surveys were conducted in 1991, 1993, 1997, 2000, 2004, 2006, 2009, 2011, and 2015. A multistage, stratified, random cluster was used to draw samples from the participating provinces (Beijing, Chongqing, Guangxi, Guizhou, Heilongjiang, Henan, Hubei, Hunan, Jiangsu, Liaoning, Shanxi, Shandong, Shanghai, Yunnan, and Zhejiang).

**Fig 1:**
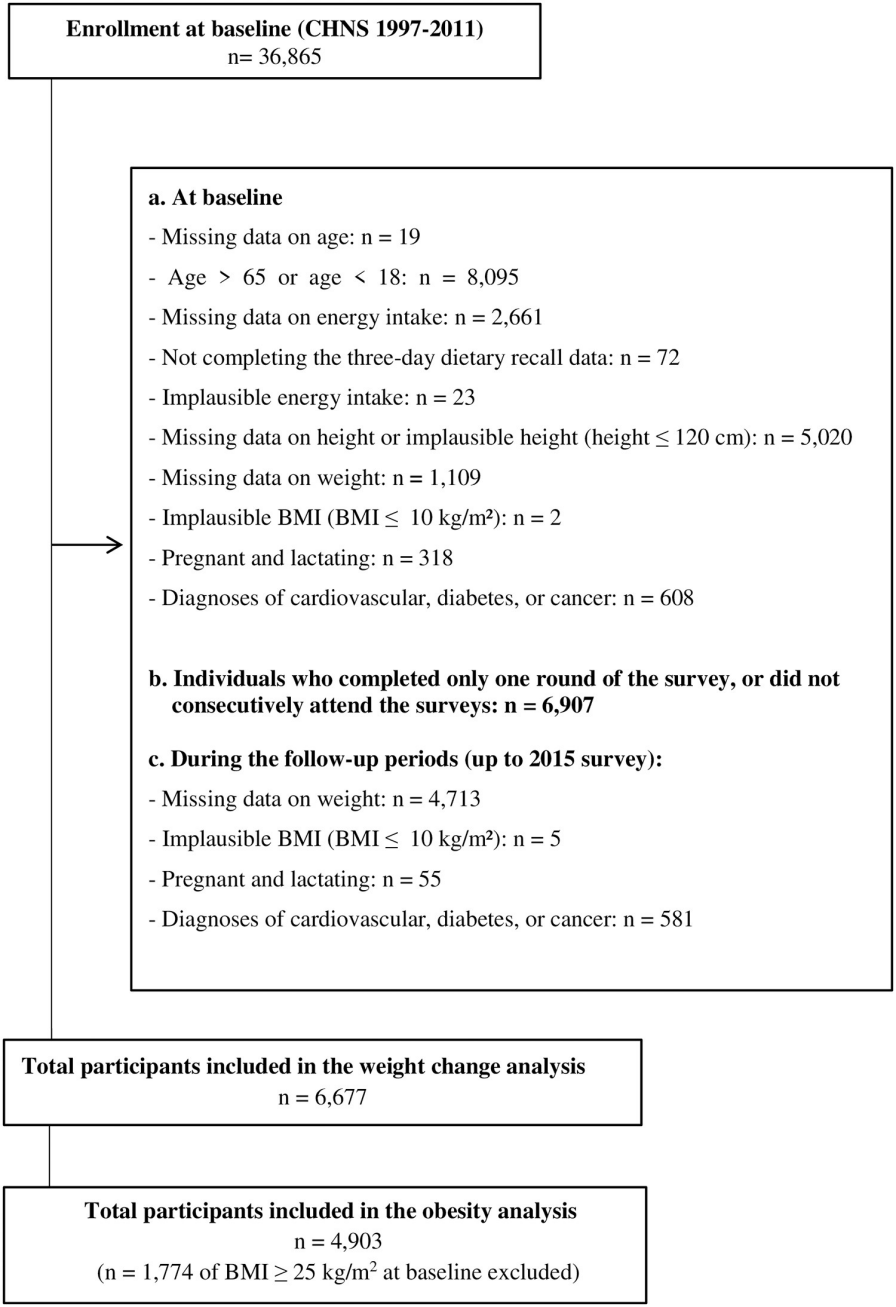
Flowchart of participants included in the study (CHNS 1997–2015).

In this study, our analysis initially included 28,751 participants aged 18–65 years from the 1997–2011 surveys, which had information of food names corresponding to the food codes. We excluded participants at baseline who had missing information on energy intake or did not complete the three-day dietary recall data (n = 2,733), reported implausible energy intake (outside the range of log-transformed mean energy intake ± 3 standard deviation) (n = 23), had missing or extreme anthropometric data (BMI ≤ 10 kg/m^2^ or height ≤ 120 cm) (n = 6,131), were currently pregnant or lactating (n = 318), or had been previously diagnosed with cardiovascular disease, diabetes, or cancer (n = 608) (Fig 1). We also excluded participants who completed only one round of the survey or did not consecutively attend the surveys (n = 6,907). We further excluded participants who had missing anthropometric data or a BMI of less than or equal to 10 kg/m^2^ (n = 4,718), were currently pregnant or lactating (n = 55), or had a history of cardiovascular disease, diabetes, or cancer (n = 581) during the follow-up periods.

As a result, a total of 6,677 participants (3,771 women and 2,906 men) were included in this analysis. When we examined the association between dietary patterns and obesity risk, we included 4,903 participants (2,771 women and 2,132 men) after excluding those with a BMI of 25 kg/m^2^ or greater at baseline.

The study was approved by the institutional review committees of the University of North Carolina (USA) and the National Institute of Nutrition and Food Safety (China). A written informed consent was obtained from all participants. Additional information regarding the ethical, cultural, and scientific considerations specific to inclusivity in global research is included in the S1 Checklist.

### Outcome definitions

Two major outcomes were defined: (1) incident obesity since baseline, defined as BMI ≥ 25 kg/m^2^ by the Asian-Pacific guidelines [18]; and (2) average weight change per five years, which was calculated as the most recently recorded weight minus the baseline weight divided by the follow-up time in years multiplied by five.

### Dietary and anthropometric assessments at baseline

Individual dietary intake was collected over three days, randomly allocated from Monday to Sunday, through a 24-hour dietary recall at the individual level. This was combined with the individual consumption of edible oils and condiments from a household inventory to improve the accuracy of the dietary assessment method as described in detail elsewhere [19, 20]. Individual consumption of cooking oils and condiments was estimated from the household dietary data by the ratio of his or her energy intake to the energy intake of all family members [21]. Nutrient intake was calculated by using corresponding versions of the Chinese Food Composition Table [22, 23].

Using standard protocols and uniform equipment (height: SECA Stadiometer 206; weight: electronic weight scale), height and body weight were directly measured at each visit to the nearest 0.1 cm and 0.1 kg, respectively, with the guidance of trained research assistants. When measuring height and body weight, participants were asked to wear light clothing and to take off their shoes. BMI was calculated as weight divided by the square of height (kg/m^2^).

### Covariate assessment

Trained interviewers administered a structured questionnaire to collect information, including sociodemographic and lifestyle factors in the CHNS. The region was classified into the Northern region (Liaoning, Heilongjiang, and Beijing), Eastern region (Jiangsu, Shandong, and Shanghai), Western region (Chongqing, Guangxi, and Guizhou), and Central region (Henan, Hubei, and Hunan). Urbanization was measured using a validated urbanization index developed by Jones-Smith and Popkin [24]. Alcohol drinking (g/d) during the past year was calculated by summing the daily ethanol intake of all alcoholic beverages included in the questionnaire. The ethanol content per serving of alcoholic beverages used to calculate each beverage’s daily ethanol intake was based on the Chinese Food Composition Table [23]. The pack-years of smoking were calculated by the dose and duration each participant smoked. The level of leisure physical activity expressed as metabolic equivalent tasks (METs) was calculated by multiplying the average time spent per week by its specific MET values [25, 26]. Household income was evaluated by household income per capita inflated to 2015. Because the information on whether participants were postmenopausal or not was only investigated in 1993, women were considered postmenopausal if they reported postmenopausal in the 1993 survey or were 50 years of age and older based on the reported median age of natural menopause among Chinese women [27]. The proportion of missing data for each variable was below 5%. We assigned the missing values to the most frequent category for categorical variables and the median value for continuous variables.

### Statistical analysis

The individual food items were aggregated into 35 groups of foods of similar nutrient content, biological origin, and culinary usage (S1 Table). We used baseline dietary intake data to identify dietary patterns. We conducted a principal component analysis using the PROC FACTOR command in SAS (Statistical Analysis System, SAS Institute, Cary, NC, USA) to drive underlying dietary patterns. We used eigenvalue (> 1.5), a scree plot, and factor interpretability to determine the number of factors to be retained [28]. The three retained factors were rotated with the varimax rotation to minimize the correlation within the factors and to increase interpretability. Factor loadings were extracted for each of the food groups across the retained factors. The dietary factors were labeled according to the food groups with a factorial load ≥ 0.2 or ≤ -0.2. For each individual, factor scores were assigned by summing the standardized intake of food groups weighted by their factor loadings. We used the residual method to adjust for total energy intake on the factor scores [29].

Each dietary pattern score at baseline was categorized into quintiles, and the first quintile was used as the reference for all analyses. The baseline demographic characteristics of participants were expressed as mean ± standard deviation (SD) for continuous variables and numbers and percentages for categorical variables according to the quintiles of each dietary pattern score.

The multivariable Cox proportional hazards regression model was used to assess the relative risks (RRs) and 95% confidence intervals (CIs) of obesity associated with each dietary pattern score [30]. Person-time of follow-up for each participant was calculated from the date of recruitment to the date when participants were diagnosed with obesity, the date of death, or the date of last participation, whichever occurred first. Multiple linear regressions were used to calculate the least-squares means (LS-means) with 95% confidence intervals (CIs) for the association between each dietary pattern score and average body weight change (kg/5 years) [31].

For both the obesity and weight change analysis, we adjusted for age (continuous, years), sex (men, women), energy intake (continuous, kcal/d), marital status (never married, married, and divorced), physical activity (0, 0 <- 18, 18 <- 36, and > 36 MET-h/wk), smoking status (0, 0 <- 10, 10 <- 20, 20 <- 30, and > 30 pack-years), alcohol drinking (0, 0 <- 6, 6 <- 12, 12 <- 24, and > 24 g/d), region (Northern, Eastern, Western, and Central regions), education level (illiteracy, primary school, junior high school, and high school or higher), household income per capita inflated to 2015 values (tertile, RMB), and urbanization index (tertile). For weight change analysis, we additionally adjusted for baseline body weight (continuous, kg). Additional adjustment for menopause status did not change the estimates for women, and thus, menopause status was not included in the final model. A test for linear trend was performed by assigning the median value of each quintile to the corresponding participant and treating the value as a continuous variable. We estimated RRs (95% CIs) for obesity for a one-unit increase in each score. We evaluated the potential modification in the subgroup analyses of age (< 42 or ≥ 42, median) and region (Southern region or Northern region). P for interaction was tested using the likelihood ratio test or Wald test.

We further conducted several sensitivity analyses to see whether the overall associations would persist. In the association between dietary patterns and obesity (BMI ≥ 25 kg/m^2^), 1) we treated the outcome as BMI ≥ 23 kg/m^2^. In the association between dietary patterns and average body weight change (kg/5 years), 1) we censored the participants who reached over 65 years of age during the follow-up period to minimize bias from age-related weight loss, 2) we restricted our analyses to the participants with BMI < 30 kg/m^2^ at baseline, 3) we excluded the participants with less than three years of follow-up, or 4) we truncated change in weight at the 1^st^ and 99^th^ percentiles to minimize the influence of outliers.

All statistical tests with a two-sided p < 0.05 were considered statistically significant. All statistical analyses were performed using SAS version 9.4 (SAS Institute, Cary, NC, USA).

## Results

Three major dietary patterns were identified by factor analysis, which explained 17% of the variance (Table 1). The Westernized dietary pattern was characterized by high intakes of high-fat dairy products, fruits and fresh juices, buns and bread, instant foods, eggs, sweets and snacks, deep-fried products, coarse grain, Western-style fast foods, nuts and seeds, and sugar-sweetened beverages combined with low intakes of rice, animal oil, preserved vegetables, and leafy green vegetables. The traditional Chinese dietary pattern was characterized by high intakes of rice, red meats, fish, poultry, organ meats, and leafy green vegetables combined with low intakes of wheat, corn, coarse grain, and buns and bread. The high-starch plant-based dietary pattern was characterized by high intakes of legumes, other vegetables, starch vegetables, orange-red vegetables, fruits and fresh juices, and preserved vegetables combined with low intakes of leafy green vegetables, cruciferous vegetables, and wheat products.

**Table 1:** Factor loadings^a^ for food groups of the three dietary patterns identified (n = 6,677).

Food groups	Westernized dietary pattern	Traditional Chinese dietary pattern	High-starch plant-based dietary pattern
High-fat dairy products	0.46	0.21	
Fruits and fresh juices	0.45	0.21	0.24
Buns and breads	0.43	-0.25	
Instant foods	0.42		
Eggs and its products	0.42		
Sweets and Snacks	0.36		
Deep-fried products	0.34		
Low-fat dairy products	0.33		
Coarse grain	0.28	-0.27	
Western-style fast foods	0.25		
Nuts and seeds	0.25		
Sugar-sweetened beverages	0.23		
Legumes			
Coffee and tea			
Preserved fruit			
Preserved vegetables	-0.26		0.21
Animal oils	-0.34		
Rice and its products	-0.58	0.53	
Red meats		0.51	
Fish and seafood		0.47	
Poultry		0.38	
Organ meats		0.24	
Alcoholic beverages			
Others			
Corn and its products		-0.43	
Wheat and its products		-0.61	-0.20
Legumes			0.61
Other vegetables			0.55
Starch vegetable and its products			0.46
Orange-red vegetables			0.27
Vegetable oils			
Processed meats			
Condiments and spice			
Cruciferous vegetables			-0.23
Leafy green vegetables	-0.21	0.21	-0.48

^a^Factor loadings with absolute values < 0.20 are not presented for simplicity.

### Characteristics of participants

The mean (± SD) age of the 6,677 participants was 43.11 ± 11.84 years. Their mean average weight change per five years was +1.15 kg. A total of 1,326 new cases of obesity were documented during a mean of 8.62 years (42276.55 person-years) of follow-up. As shown in Table 2, we found increasing levels of weight, BMI, leisure physical activity, and household income with increasing scores of the Westernized dietary pattern. A higher proportion of participants with high Westernized dietary pattern scores lived in the Northern region and had higher levels of urbanization and education compared to those with lower scores. Higher protein and fat percent energy intake were observed in participants with high Westernized dietary pattern scores compared to those with lower scores. Participants with high traditional Chinese dietary pattern scores tended to have higher levels of leisure physical activity, and household income compared to those with lower scores. Contrary to the Westernized dietary pattern, a high proportion of participants who followed traditional Chinese dietary patterns lived in the Southern region. Higher levels of urbanization and education were observed in participants with high traditional Chinese dietary pattern scores. Participants in the highest quintile of traditional Chinese dietary pattern scores tended to consume a lower percent energy intake from carbohydrates, but higher percent energy intake from protein and fat compared to those in the lowest quintile. For the high-starch plant-based dietary pattern, participants with high high-starch plant-based dietary pattern scores tended to engage in leisure physical activity and live in the Northern region.

**Table 2:** Baseline characteristics according to quintiles of dietary patterns in men and women combined.

	Westernized dietary pattern			Traditional Chinese dietary pattern			High-starch plant-based dietary pattern		
	Quintile 1	Quintile 3	Quintile 5	Quintile 1	Quintile 3	Quintile 5	Quintile 1	Quintile 3	Quintile 5
No. of participants	1335	1335	1335	1335	1335	1335	1335	1335	1335
Age (y)	43.26 ± 11.52	42.54 ± 11.56	43.61 ± 12.45	42.14 ± 11.57	43.50 ± 11.78	43.22 ± 12.00	43.19 ± 11.79	42.95 ± 12.09	43.17 ± 11.64
Weight (kg)	55.26 ± 9.21	61.31 ± 10.33	65.44 ± 12.33	61.44 ± 10.22	59.39 ± 12.04	61.93 ± 11.22	59.59 ± 11.17	60.85 ± 11.38	61.63 ± 11.33
BMI (kg/m^2^)	22.11 ± 2.88	23.39 ± 3.27	24.00 ± 3.63	23.31 ± 3.18	23.05 ± 3.65	23.30 ± 3.34	22.96 ± 3.27	23.32 ± 3.44	23.31 ± 3.35
Leisure physical activity (MET-h/wk)	1.65 ± 8.16	4.80 ± 16.30	12.34 ± 28.64	2.82 ± 12.26	6.41 ± 20.76	8.65 ± 23.46	4.96 ± 19.78	6.34 ± 18.92	6.88 ± 22.47
Alcohol drinking (g/d)	8.19 ± 22.28	9.15 ± 23.97	9.14 ± 23.79	8.49 ± 21.59	7.99 ± 22.49	10.22 ± 24.20	9.49 ± 24.48	7.70 ± 21.77	10.69 ± 25.64
Household income per capita (1,000 RMB) ^a^	5.40 ± 6.33	9.38 ± 10.72	18.92 ± 18.91	6.53 ± 7.01	11.06 ± 14.21	14.96 ± 18.29	8.47 ± 11.38	11.72 ± 13.45	10.86 ± 12.79
**Region** ^b^									
North	226 (16.9)	550 (41.2)	896 (67.1)	1133 (84.9)	421 (31.5)	188 (14.1)	460 (34.5)	493 (36.9)	809 (60.6)
South	1109 (83.1)	785 (58.8)	439 (32.9)	202 (15.1)	914 (68.5)	1147 (85.9)	875 (65.5)	842 (63.1)	526 (39.4)
**Urbanization index** ^c^									
Low	662 (49.6)	322 (24.1)	80 (6.0)	558 (41.8)	313 (23.4)	202 (15.1)	492 (36.9)	265 (19.9)	409 (30.6)
Median	517 (38.7)	425 (31.8)	237 (17.8)	460 (34.5)	397 (29.7)	382 (28.6)	452 (33.9)	395 (29.6)	393 (29.4)
High	156 (11.7)	588 (44.0)	1018 (76.3)	317 (23.7)	625 (46.8)	751 (56.3)	391 (29.3)	675 (50.6)	533 (39.9)
**Education level** ^d^									
Illiteracy	410 (30.7)	211 (15.8)	68 (5.1)	312 (23.4)	231 (17.3)	130 (9.7)	316 (23.7)	198 (14.8)	217 (16.3)
Primary or junior high school	760 (56.9)	714 (53.5)	454 (34.0)	732 (54.8)	632 (47.3)	619 (46.4)	679 (50.9)	626 (46.9)	686 (51.4)
High school or vocational-technical school	123 (9.2)	297 (22.2)	494 (37.0)	228 (17.1)	309 (23.1)	406 (30.4)	250 (18.7)	344 (25.8)	288 (21.6)
Some college or above	14 (1.0)	97 (7.3)	312 (23.4)	43 (3.2)	145 (10.9)	174 (13.0)	67 (5.0)	153 (11.5)	133 (10.0)
**Smoking status** ^d^									
Non-smoker	940 (70.4)	926 (69.4)	962 (72.1)	924 (69.2)	990 (74.2)	864 (64.7)	928 (69.5)	962 (72.1)	910 (68.2)
Former smoker	14 (1.0)	24 (1.8)	34 (2.5)	14 (1.0)	20 (1.5)	30 (2.2)	18 (1.3)	32 (2.4)	30 (2.2)
Current smoker	371 (27.8)	379 (28.4)	336 (25.2)	385 (28.8)	317 (23.7)	435 (32.6)	376 (28.2)	337 (25.2)	392 (29.4)
**Daily nutrient intake**									
Energy intake (kcal/d)	2189 ± 695.4	2057 ± 674.1	2141 ± 650.9	2252 ± 655.4	1912 ± 612.1	2308 ± 685.5	2295 ± 669.0	1959 ± 637.4	2241 ± 673.8
Carbohydrate intake (% of energy intake)	63.88 ± 12.34	54.84 ± 13.14	48.99 ± 10.86	63.97 ± 10.63	55.74 ± 12.58	47.71 ± 12.39	58.25 ± 13.72	54.08 ± 13.14	57.54 ± 12.55
Protein intake (% of energy intake)	11.37 ± 2.59	13.07 ± 3.15	14.92 ± 3.68	12.09 ± 2.17	12.90 ± 3.36	15.07 ± 3.82	12.78 ± 2.96	13.41 ± 3.63	12.85 ± 3.39
Fat intake (% of energy intake)	24.31 ± 12.11	31.27 ± 12.62	35.10 ± 10.47	23.41 ± 10.51	30.80 ± 12.14	36.11 ± 11.66	27.96 ± 12.56	31.80 ± 12.26	28.78 ± 11.71

Abbreviations: BMI, body mass index (calculated as weight in kilograms divided by height in meters squared); MET-h, metabolic task equivalent-hours; RMB, Ren Min Bi

Values are mean ± SD for continuous variable and % for categorical variables.

^a^Total net household income inflated to 2015.

^b^Northern region (Beijing, Liaoning, Heilongjiang, Shandong, Henan); Southern region (Shanghai, Jiangsu, Hubei, Hunan, Guangxi, Guizhou, Chongqing).

^c^Urbanization index was developed by Jones-Smith and Popkin, including 12 components to capture community-level physical, social, cultural, and economic environments.

^d^A few participants did not have data on these variables.

### Dietary patterns and obesity

An increase in the Westernized dietary pattern score was significantly associated with an increased risk of obesity with multivariate adjustments for lifestyle factors, sex, age, and energy intake (Table 3). The RR (95% CI) comparing the top and bottom quintiles was 2.13 (1.78–2.56; P for trend < 0.001), which was slightly attenuated to 1.57 (1.26–1.95) after further adjusting for socioeconomic status (SES)-related variables. A per one-unit score increase was associated with an 18% higher risk of obesity (RR, 1.18; 95% CI, 1.10–1.26). In contrast, we observed an inverse association between the traditional Chinese dietary pattern and obesity, RRs (95% CI) compared to the bottom quintile were 0.78 (0.66–0.92) for the 2^nd^ quintile, 0.83 (0.69–0.98) for the 3^rd^ quintile, 0.80 (0.66–0.96) for the 4^th^ quintile, and 0.84 (0.70–1.01) for top quintile (P for trend = 0.045). RR (95% CI) for a per one-unit increase in the traditional Chinese dietary pattern score was 0.95 (95%CI, 0.89–1.01). For the high-starch plant-based dietary pattern score, no significant association was observed.

**Table 3:** Relative risk (RR)s of incident obesity (BMI ≥ 25kg/m^2^) according to quintiles of dietary patterns in men and women combined.

	Total population (n = 4,903)						
	Quintile 1	Quintile 2	Quintile 3	Quintile 4	Quintile 5	P for trend^d^	Per one unit increase
**Westernized dietary pattern**							
Person-years of follow up	11457.89	9552.61	8618.44	7216.31	5431.29		
Cases, No.	269	267	278	278	234		
Model 1^a^	1.00	1.24 (1.05–1.47)	1.47 (1.24–1.74)	1.83 (1.54–2.16)	2.14 (1.79–2.56)	< .001	1.30 (1.23–1.37)
Model 2^b^	1.00	1.25 (1.06–1.49)	1.47 (1.24–1.74)	1.83 (1.55–2.18)	2.13 (1.78–2.56)	< .001	1.30 (1.23–1.37)
Model 3^c^	1.00	1.15 (0.97–1.37)	1.25 (1.04–1.50)	1.48 (1.22–1.79)	1.57 (1.26–1.95)	< .001	1.18 (1.10–1.26)
**Traditional Chinese dietary pattern**							
Person-years of follow up	9265.07	8428.55	8543.22	8432.69	7607.03		
Cases, No.	343	257	254	236	236		
Model 1^a^	1.00	0.81 (0.69–0.95)	0.81 (0.68–0.95)	0.79 (0.67–0.93)	0.89 (0.76–1.05)	0.050	0.97 (0.92–1.02)
Model 2^b^	1.00	0.80 (0.68–0.94)	0.80 (0.68–0.94)	0.78 (0.66–0.92)	0.87 (0.74–1.03)	0.027	0.96 (0.91–1.01)
Model 3^c^	1.00	0.78 (0.66–0.92)	0.83 (0.69–0.98)	0.80 (0.66–0.96)	0.84 (0.70–1.01)	0.045	0.95 (0.89–1.01)
**High-starch plant-based dietary pattern**							
Person-years of follow up	10021.34	8185.79	7834.49	7797.53	8437.40		
Cases, No.	289	261	240	235	301		
Model 1^a^	1.00	1.10 (0.93–1.31)	1.05 (0.88–1.24)	1.00 (0.84–1.19)	1.14 (0.97–1.34)	0.249	1.00 (0.95–1.05)
Model 2^b^	1.00	1.10 (0.93–1.30)	1.04 (0.87–1.23)	0.99 (0.83–1.18)	1.14 (0.97–1.34)	0.262	1.00 (0.95–1.05)
Model 3^c^	1.00	1.02 (0.86–1.22)	0.93 (0.78–1.11)	0.89 (0.74–1.06)	1.03 (0.86–1.22)	0.964	0.98 (0.93–1.03)

Abbreviations: RR, relative risk; CI, confidence interval

^a^Model 1 was adjusted for sex (men, women), age (continuous, years), and energy intake (continuous, kcal/d).

^b^Model 2 was adjusted for sex (men, women), age (continuous, years), energy intake (continuous, kcal/d), physical activity (0, 0 <- 18, 18 <- 36, and > 36 MET-h/wk), smoking status (0, 0 <- 10, 10 <- 20, 20 <- 30, and > 30 pack-years), and alcohol drinking (0, 0 <- 6, 6 <- 12, 12 <- 24, and > 24 g/d).

^c^Model 3 was adjusted for sex (men, women), age (continuous, years), energy intake (continuous, kcal/d), physical activity (0, 0 <- 18, 18 <- 36, and > 36 MET-h/wk), smoking status (0, 0 <- 10, 10 <- 20, 20 <- 30, and > 30 pack-years), alcohol drinking (0, 0 <- 6, 6 <- 12, 12 <- 24, and > 24 g/d), region (Northern region, Eastern region, Western region, and Central region), education level (illiteracy, primary school, junior high school, and high school or higher), marital status (never married, married, and divorced), household income per capita inflated to 2015 (tertile, RMB), and urbanization index (tertile).

^d^P for trend was calculated using the median value of each quintile category as a continuous variable.

When we examined the association in men and women separately, we found an increased risk of obesity with the Westernized dietary pattern in both men and women (S2 and S3 Tables). For the traditional Chinese dietary pattern, we found a trend toward an inverse association with obesity risk in men but attenuation in risk with adjustment for SES variables in women. For the high-starch plant-based dietary pattern, we did not find clear associations.

The Westernized dietary pattern increased the risk of obesity in both Southern and Northern regions or in both young and old ages (Table 4).

**Table 4:** Relative risk (RR)s^a^ of incident obesity (BMI ≥ 25kg/m^2^) according to quintiles of dietary patterns by age and region.

	**Westernized dietary pattern**						
	**Quintile 1**	**Quintile 2**	**Quintile 3**	**Quintile 4**	**Quintile 5**	**P for trend**	**P for interaction**
**Age, years**							0.037
**< 42 years**							
No. of cases / person-years	133 / 5272.65	138 / 4423.17	152 / 4676.44	148 / 3739.65	103 / 2622.21		
RR (95% CI)	1.00	1.24 (0.96–1.58)	1.20 (0.93–1.55)	1.55 (1.18–2.03)	1.62 (1.18–2.23)	< .001	
**≥ 42 years**							
No. of cases / person-years	136 / 6185.24	129 / 5129.45	126 / 3942.01	130 / 3476.67	131 / 2809.08		
RR (95% CI)	1.00	1.10 (0.85–1.41)	1.30 (1.00–1.69)	1.46 (1.10–1.92)	1.58 (1.16–2.14)	< .001	
**Region**							0.917
**North**							
No. of cases / person-years	40 / 1490.17	69 / 2164.54	134 / 3716.16	192 / 4296.34	164 / 3770.64		
RR (95% CI)	1.00	1.18 (0.80–1.75)	1.37 (0.96–1.96)	1.66 (1.17–2.34)	1.55 (1.08–2.24)	0.015	
**South**							
No. of cases / person-years	229 / 9967.73	198 / 7388.07	144 / 4902.28	86 / 2919.98	70 / 1660.65		
RR (95% CI)	1.00	1.16 (0.95–1.41)	1.19 (0.95–1.49)	1.17 (0.88–1.54)	1.58 (1.14–2.20)	0.011	
	**Traditional Chinese dietary pattern**						
	**Quintile 1**	**Quintile 2**	**Quintile 3**	**Quintile 4**	**Quintile 5**	**P for trend**	**P for interaction**
**Age, years**							0.848
**< 42 years**							
No. of cases / person-years	191 / 4744.05	130 / 4284.96	116 / 4048.72	117 / 3888.33	120 / 3768.05		
RR (95% CI)	1.00	0.73 (0.57–0.93)	0.73 (0.57–0.94)	0.82 (0.63–1.07)	0.83 (0.64–1.08)	0.162	
**≥ 42 years**							
No. of cases / person-years	152 / 4521.01	127 / 4143.59	138 / 4494.50	119 / 4544.35	116 / 3838.98		
RR (95% CI)	1.00	0.85 (0.66–1.09)	0.94 (0.73–1.21)	0.78 (0.61–1.01)	0.83 (0.63–1.08)	0.122	
**Region**							0.881
**North**							
No. of cases / person-years	287 / 7189.90	147 / 4272.40	79 / 1963.96	49 / 1197.85	37 / 813.74		
RR (95% CI)	1.00	0.76 (0.62–0.94)	0.87 (0.67–1.13)	0.92 (0.67–1.26)	0.93 (0.65–1.34)	0.272	
**South**							
No. of cases / person-years	56 / 2075.17	110 / 4156.15	175 / 6579.26	187 / 7234.84	199 / 6793.29		
RR (95% CI)	1.00	0.97 (0.69–1.35)	0.99 (0.73–1.36)	0.96 (0.70–1.32)	1.02 (0.75–1.40)	0.799	
	**High-starch plant-based dietary pattern**						
	**Quintile 1**	**Quintile 2**	**Quintile 3**	**Quintile 4**	**Quintile 5**	**P for Trend**	**P for interaction**
**Age, years**							0.582
**< 42 years**							
No. of cases / person-years	144 / 4551.15	123 / 3955.39	128 / 4134.38	116 / 3934.66	163 / 4158.54		
RR (95% CI)	1.00	0.95 (0.74–1.22)	0.90 (0.70–1.15)	0.86 (0.66–1.11)	1.10 (0.86–1.41)	0.437	
**≥ 42 years**							
No. of cases / person-years	145 / 5470.19	138 / 4230.39	112 / 3700.11	119 / 3862.87	138 / 4278.87		
RR (95% CI)	1.00	1.10 (0.86–1.40)	0.94 (0.73–1.22)	0.90 (0.70–1.16)	0.93 (0.72–1.19)	0.319	
**Region**							0.202
**North**							
No. of cases / person-years	114 / 3085.36	99 / 2270.10	96 / 2620.59	117 / 3149.65	173 / 4312.15		
RR (95% CI)	1.00	1.09 (0.82–1.45)	0.88 (0.66–1.18)	0.85 (0.65–1.13)	0.94 (0.73–1.21)	0.431	
**South**							
No. of cases / person-years	175 / 6935.98	162 / 5915.69	144 / 5213.91	118 / 4647.88	128 / 4125.26		
RR (95% CI)	1.00	1.02 (0.82–1.27)	0.98 (0.78–1.23)	0.93 (0.73–1.18)	1.10 (0.87–1.39)	0.638	

Abbreviations: RR, relative risk; CI, confidence interval

^a^Models were adjusted for sex (men, women), age (continuous, years), energy intake (continuous, kcal/d), physical activity (0, 0 <- 18, 18 <- 36, and > 36 MET-h/wk), smoking status (0, 0 <- 10, 10 <- 20, 20 <- 30, and > 30 pack-years), alcohol drinking(0, 0 <- 6, 6 <- 12, 12 <- 24, and > 24 g/d), region (Northern region, Eastern region, Western region, and Central region), education level (illiteracy, primary school, junior high school, and high school or higher), marital status (never married, married, and divorced), household income per capita inflated to 2015 (tertile, RMB), and urbanization index (tertile), except the corresponding subgroup variates.

A similar association between the Westernized dietary pattern and obesity risk was observed when the outcome was overweight or obesity (BMI ≥ 23 kg/m^2^) (S4 Table). However, the association between the traditional Chinese dietary pattern and obesity risk did not persist.

### Dietary patterns and average weight change over five years

Higher Westernized dietary pattern scores were associated with increased levels of weight gain (Table 5). The LS-means of weight change (kg/5 years) were 1.13 (0.39–1.87) in the top quintile and 1.73 (0.98–2.47; P for trend = 0.036) in the bottom quintile. No significant association was observed for the traditional Chinese dietary pattern or the high-starch plant-based dietary pattern. Similar results were observed in men, but the association for the Westernized dietary pattern was unclear in women (S5 and S6 Tables).

**Table 5:** Least-squares means of body weight change (kg/5 years) according to quintiles of dietary patterns in men and women combined.

	Total population (n = 6,677)					
	Quintile 1	Quintile 2	Quintile 3	Quintile 4	Quintile 5	P for trend^d^
**Westernized dietary pattern**						
Weight change, median (kg/5 years)	1.03	1.07	0.98	1.02	0.88	
Model 1^a^	1.39 (0.98–1.81)	0.90 (0.49–1.31)	1.22 (0.81–1.63)	1.43 (1.02–1.84)	0.88 (0.47–1.29)	0.312
Model 2^b^	2.06 (1.30–2.82)	1.64 (0.91–2.37)	2.08 (1.35–2.82)	2.33 (1.59–3.08)	1.76 (1.00–2.53)	0.776
Model 3^c^	1.13 (0.39–1.87)	1.01 (0.30–1.72)	1.73 (1.02–2.44)	2.04 (1.32–2.76)	1.73 (0.98–2.47)	0.036
**Traditional Chinese dietary pattern**						
Weight change, median (kg/5 years)	1.11	0.99	0.91	0.99	1.02	
Model 1^a^	1.21 (0.80–1.62)	1.39 (0.98–1.81)	0.99 (0.58–1.41)	1.06 (0.65–1.47)	1.17 (0.75–1.58)	0.572
Model 2^b^	2.05 (1.30–2.80)	2.16 (1.42–2.90)	1.75 (1.02–2.48)	1.86 (1.12–2.60)	2.01 (1.27–2.75)	0.629
Model 3^c^	1.85 (1.13–2.58)	1.76 (1.04–2.47)	1.18 (0.47–1.89)	1.20 (0.48–1.91)	1.55 (0.84–2.27)	0.109
**High-starch plant-based dietary pattern**						
Weight change, median (kg/5 years)	0.90	0.84	1.15	1.06	1.05	
Model 1^a^	0.93 (0.52–1.35)	0.94 (0.53–1.36)	1.16 (0.75–1.58)	1.51 (1.10–1.92)	1.27 (0.86–1.68)	0.092
Model 2^b^	1.70 (0.97–2.44)	1.78 (1.04–2.52)	1.97 (1.24–2.70)	2.32 (1.59–3.06)	2.00 (1.26–2.74)	0.163
Model 3^c^	1.34 (0.63–2.06)	1.33 (0.61–2.05)	1.52 (0.81–2.23)	1.76 (1.05–2.47)	1.57 (0.85–2.29)	0.255

Abbreviations: LS-mean, least-squares mean; CI, confidence interval

^a^Model 1 was adjusted for sex (men, women), age (continuous, years), and energy intake (continuous, kcal/d).

^b^Model 2 was adjusted for sex (men, women), age (continuous, years), energy intake (continuous, kcal/d), physical activity (0, 0 <- 18, 18 <- 36, and > 36 MET-h/wk), smoking status (0, 0 <- 10, 10 <- 20, 20 <- 30, and > 30 pack-years), alcohol drinking(0, 0 <- 6, 6 <- 12, 12 <- 24, and > 24 g/d), region (Northern region, Eastern region, Western region, and Central region), education level (illiteracy, primary school, junior high school, and high school or higher), marital status (never married, married, and divorced), household income per capita inflated to 2015 (tertile, RMB), and urbanization index (tertile).

^c^Model 3 was adjusted for baseline body weight (continuous, kg) in addition to variables in Model 2.

^d^P for trend was calculated using the median value of each quintile category as a continuous variable.

In the Southern region, where the urbanization index is higher than that in the Northern region in this study, there was a more pronounced association between the Westernized dietary pattern and weight gain (P for trend < 0.001) compared to the Northern region (Table 6). When stratified by age, the association between the Westernized dietary pattern and weight gain was limited to young adults.

**Table 6:** Least-squares means^a^ of body weight change (kg/5 years) according to quintiles of dietary patterns by age and region.

	**Westernized dietary pattern**						
	**Quintile 1**	**Quintile 2**	**Quintile 3**	**Quintile 4**	**Quintile 5**	**P for trend**	**P for interaction**
**Age, years**							0.767
**< 42 years**							
N	587	616	636	622	572		
LS-mean (95% CI)	2.63 (1.28–3.97)	2.57 (1.26–3.88)	2.81 (1.50–4.12)	3.82 (2.50–5.13)	3.57 (2.19–4.95)	0.015	
**≥ 42 years**							
N	748	720	699	714	763		
LS-mean (95% CI)	0.25 (-0.87–1.36)	0.02 (-1.07–1.10)	1.18 (0.08–2.29)	0.80 (-0.30–1.91)	0.48 (-0.64–1.61)	0.612	
**Region**							0.049
**North**							
N	226	348	550	793	896		
LS-mean (95% CI)	0.62 (-0.95–2.20)	0.78 (-0.63–2.19)	1.30 (-0.01–2.60)	1.14 (-0.11–2.38)	0.50 (-0.74–1.74)	0.400	
**South**							
N	1109	988	785	543	439		
LS-mean (95% CI)	1.48 (0.68–2.28)	1.37 (0.58–2.15)	2.00 (1.17–2.82)	2.50 (1.62–3.39)	2.86 (1.90–3.81)	< .001	
	**Traditional Chinese dietary pattern**						
	**Quintile 1**	**Quintile 2**	**Quintile 3**	**Quintile 4**	**Quintile 5**	**P for trend**	**P for interaction**
**Age, years**							0.650
**< 42 years**							
N	648	615	607	586	577		
LS-mean (95% CI)	3.36 (2.03–4.70)	3.15 (1.83–4.47)	2.72 (1.41–4.02)	3.00 (1.67–4.34)	3.03 (1.71–4.36)	0.388	
**≥ 42 years**							
N	687	721	728	750	758		
LS-mean (95% CI)	0.82 (-0.30–1.95)	0.86 (-0.24–1.95)	0.16 (-0.92–1.24)	0.09 (-1.01–1.18)	0.64 (-0.46–1.74)	0.297	
**Region**							0.400
**North**							
N	1133	794	421	277	188		
LS-mean (95% CI)	0.88 (-0.32–2.08)	1.30 (0.07–2.54)	0.29 (-1.07–1.65)	0.19 (-1.28–1.66)	1.34 (-0.30–2.98)	0.655	
**South**							
N	202	542	914	1059	1147		
LS-mean (95% CI)	1.79 (0.63–2.96)	1.76 (0.88–2.64)	1.75 (0.96–2.54)	1.82 (1.03–2.60)	2.05 (1.28–2.83)	0.379	
	**High-starch plant-based dietary pattern**						
	**Quintile 1**	**Quintile 2**	**Quintile 3**	**Quintile 4**	**Quintile 5**	**P for Trend**	**P for interaction**
**Age, years**							0.487
**< 42 years**							
N	586	606	619	603	619		
LS-mean (95% CI)	2.81 (1.48–4.14)	2.56 (1.25–3.87)	3.32 (2.01–4.63)	3.1 (1.78–4.43)	3.42 (2.10–4.74)	0.090	
**≥ 42 years**							
N	749	730	716	733	716		
LS-mean (95% CI)	0.38 (-0.72–1.47)	0.61 (-0.49–1.72)	0.28 (-0.81–1.38)	0.89 (-0.18–1.97)	0.26 (-0.84–1.36)	0.921	
**Region**							0.738
**North**							
N	460	409	493	642	809		
LS-mean (95% CI)	0.72 (-0.62–2.06)	0.32 (-1.06–1.70)	0.55 (-0.78–1.88)	1.54 (0.27–2.80)	0.77 (-0.46–2.00)	0.486	
**South**							
N	875	927	842	694	526		
LS-mean (95% CI)	1.61 (0.81–2.41)	1.91 (1.12–2.71)	2.07 (1.28–2.87)	1.69 (0.86–2.51)	2.12 (1.24–3.00)	0.280	

Abbreviations: LS-means, least-squares means; CIs, confidence intervals

^a^Models were adjusted for sex (men, women), age (continuous, years), energy intake (continuous, kcal/d), physical activity (0, 0 <- 18, 18 <- 36, and > 36 MET-h/wk), smoking status (0, 0 <- 10, 10 <- 20, 20 <- 30, and > 30 pack-years), alcohol drinking(0, 0 <- 6, 6 <- 12, 12 <- 24, and > 24 g/d), region (Northern region, Eastern region, Western region, and Central region), education level (illiteracy, primary school, junior high school, and high school or higher), marital status (never married, married, and divorced), household income per capita inflated to 2015 (tertile, RMB), urbanization index (tertile), and baseline body weight (continuous, kg), except the corresponding subgroup variates.

In four sensitivity analyses in which, we 1) censored the data at age 65 years during the follow-up, 2) excluded participants whose baseline BMIs were > 30 kg/m^2^, or 3) excluded participants whose follow-up periods were less than three years, or 4) participants who were in the 1^st^ or 99^th^ percentile of weight change (S7–S10 Tables), the point estimates were in the same direction as the estimates observed in the main analysis.

## Discussion

We identified three major dietary patterns, Westernized dietary pattern, traditional Chinese dietary pattern, and high-starch plant-based dietary pattern, in the CHNS cohort study. The Westernized dietary pattern was associated with increased obesity risk as well as body weight gain, whereas the traditional Chinese dietary pattern was marginally associated with lower obesity risk. However, no association was observed in the high-starch plant-based dietary pattern.

Similar dietary patterns identified in our study were observed in previous studies of Chinese adults [32, 33]. In a nationally representative cross-sectional study (2002 CNNHS) [33], the diet patterns of ‘Yellow Earth’, ‘Green water’, ‘Newly Affluent’, and ‘Western Adopters’ were identified. Our traditional Chinese dietary pattern was similar to the ‘Green water’ pattern, a typical traditional diet in South China, where rice is the staple food. The Westernized dietary pattern in our study resembled a combination of the ‘Yellow Earth’ dietary pattern (a typical traditional diet in North China, where wheat products are the staple food) and the ‘Western Adopters’ pattern (high in beef, lamb, dairy products, soft drinks, cakes, and juices), which represents the rapid eating behavior transition from the traditional diet to a Western diet over the last few decades [34]. In the Jiangsu Nutrition Study [32], the vegetable-rich pattern (high in whole grains, root vegetables, and fresh and pickled vegetables) was similar to the starch-rich vegetable-based dietary pattern in our study.

Our findings regarding the Westernized dietary pattern were consistent with a recent meta-analysis that showed a 65% increased risk of obesity associated with the Western dietary pattern (highly loaded with red or processed meat, refined grains, potatoes, sweets, and high-fat dairy) [10]. Several Chinese population studies conducted across various age groups have also reported that a Western-style dietary pattern characterized by high intakes of fast food, sugary beverages, and desserts was associated with an increased risk of obesity as well as body weight gain [13, 16, 35–38]. Although most of these studies were cross-sectional and limited to a specific region, our results suggest that a shift to a Westernized diet may contribute to obesity in the Chinese population.

Our results revealed an inverse association between the traditional Chinese dietary pattern and obesity occurrence. These results concur with several cross-sectional studies [11, 13, 36] and one prospective study [35] that reported a similar relationship between a traditional dietary pattern (high in rice, vegetables, and meat) and obesity in the Chinese population. Our findings may support the current Dietary Guidelines for Chinese Residents [39], which recommend adhering to a healthy Eastern dietary pattern to prevent chronic disease. According to the Dietary Guidelines, the healthy Eastern dietary pattern consists of high proportions of cereal grains, vegetables, fruits, fish, and meat, which is similar to the traditional Chinese dietary pattern examined in our study. We also found that the traditional Chinese dietary pattern was associated with a trend toward lower risk of obesity among men, but not among women. However, a study of middle-aged and elderly Chinese people in Shanghai found that a rice-staple pattern (high intake of rice, vegetables, pork, poultry, starchy roots or tubers, organ meats, processed meats, and seeds or nuts) was associated with an increased risk of obesity in men [40], contrary to our observations. Shi Z et al. [32] reported that the traditional dietary pattern (high in rice, vegetables, and pork) was inversely associated with weight gain in both men and women aged above 20 years in the Jiangsu province. However, we did not find a clear association between the traditional Chinese dietary pattern and 5-year weight gain. Further prospective studies are warranted to examine the long-term effects of adherence to the traditional Chinese dietary pattern on 5-year weight gain.

When we conducted stratified analyses, we found a stronger association between the Westernized dietary pattern and weight gain in the Southern region than in the Northern region of China. One possible explanation for the regional difference is that individuals in relatively affluent regions could have easier access to high-calorie foods (e.g., fast food and sweets) and shift to a sedentary lifestyle partly due to the increase in restaurants, screen time, and motorized transportation. Our study suggests that region-specific dietary weight management strategies are needed in China.

Several potential mechanisms explain how the Westernized dietary pattern is associated with obesity risk and weight change. The Westernized dietary pattern was characterized by high intakes of instant foods, sweets and snacks, deep-fried products, and Western-style fast foods, which generally contribute to weight gain [41, 42]. The association between the Westernized dietary pattern and obesity as well as weight gain in the present study could be partly explained by higher intakes of fat with higher scores of the Westernized dietary pattern. Fat intake was the highest among participants in the highest quintile of the Westernized dietary pattern. A long-term Chinese study reported that high fat intake was positively associated with the risk of overweight and obesity (BMI ≥ 25 kg/m^2^) because dietary fat intake contributes to a positive energy balance [43].

This study’s strengths include the use of a large nationwide sample of Chinese adults and a prospective follow-up design. We used three consecutive days of 24 h dietary recall methods to record dietary intake, which provided detailed food information. This study has several limitations as well. First, because dietary information was based on a self-reported assessment at baseline, non-differential misclassification of dietary intake was possible. Second, we did not examine changes in diet and obesity risk. Third, although we adjusted for known confounding variables in our model, residual or unknown confounding bias may still exist. Finally, our results may not be generalizable to populations in other countries.

## Conclusions

Our findings provide evidence that the Westernized dietary pattern was associated with an increased risk of obesity and weight gain. An increase in weight gain with high Westernized dietary pattern was more pronounced in the Southern than Northern regions.

## Supporting information

S1 Table Examples of food items for each food group. (DOCX)

S2 Table Relative risks of incident obesity according to quintiles of dietary patterns in men. (DOCX)

S3 Table Relative risks of incident obesity according to quintiles of dietary patterns in women. (DOCX)

S4 Table Relative risks of incident obesity (BMI ≥ 23kg/m^2^) according to quintiles of dietary patterns in men and women combined. (DOCX)

S5 Table Least-squares means of body weight change (kg/5 years) according to quintiles of dietary patterns in men. (DOCX)

S6 Table Least-squares means of body weight change (kg/5 years) according to quintiles of dietary patterns in women. (DOCX)

S7 Table Least-squares means of body weight change (kg/5 years), after censoring age of 65 years. (DOCX)

S8 Table Least-squares means of body weight change (kg/5 years) among participants who reported baseline BMI < 30 kg/m^2^. (DOCX)

S9 Table Least-squares means of body weight change (kg/5 years), after excluding participants with less than 3 years of follow-up. (DOCX)

S10 Table Least-squares means of body weight change (kg/5 years), after excluding the 1^st^ and 99^th^ percentiles of weight change. (DOCX)

S1 Checklist Checklist of inclusivity in global research. (DOCX)
