# Supplementary material for: Associations of dietary patterns with obesity and weight change for adults aged 18–65 years: Evidence from the China Health and Nutrition Survey (CHNS)
Source: PLoS One. 2023 Jan 25;18(1):e0279625. doi: 10.1371/journal.pone.0279625 (PMC9876275; doi:10.1371/journal.pone.0279625)
Supplement: S1 Table — (DOCX) [file pone.0279625.s001.docx]

| **S1 Table. Examples of food items for each food group** | |
| --- | --- |
| **Food group** | **Examples of food items** |
| 1. Rice and its products | Polished round- Grained rice, Glutinous rice, Rice noodles, Rice porridge, Rice cake, Rice ball, Rice cracker |
| 2. Wheat and its products | Wheat flour, Wheat grain, Wheat noodles, Wheat macaroni.Wheat gluten |
| 3. Buns and breads | Bun, Butter bread, Salty bread |
| 4. Deep-fried products | Deep-fried dough stick, Deep-fried cake with red bean paste and sugar, Deep-fried sweet sesame seed ball, Deep-fried rice flour doughnut, Deep-fried soybean, Deep-fried broad bean |
| 5. Corn and its products | Corn, Cornflour, Corn grits, Cornflake |
| 6. Coarse grain | Barley, Oats, Foxtail millet, Sorghum, Brown rice, Ready to eat cereals |
| 7. Starch vegetables and its products | Potato, Yam, Taro, Lotus root, Water chestnut, Cassava, Arrowhead, Potato starch, Starch noodle, Starch jelly sheet |
| 8. Leafy green vegetables | Spinach, Romaine or leaf lettuce, Bok choi, Mustard greens |
| 9. Cruciferous vegetables | Broccoli, Cabbage, Cauliflower, Brussels sprouts, Radish |
| 10. Orange-red vegetables | Carrots, Yellow (winter) squash, Sweet potato, Beetroot, Red onions, Tomato |
| 11. Other vegetables | Celery, Green pepper, Eggplant, Summer squash, Ginger, Scallion, Mushrooms, Fresh or dried seaweed, Black-moss, Pea with pods, Soybean sprouts |
| 12. Preserved vegetables | Canned tomato sauce, Preserved vegetables, Vegetables in soy sauce |
| 13. Legumes | Kidney beans, Lima beans, Soybeans, Black beans |
| 14. Legumes products | Tofu, Tofu products, Red/mung bean paste, Fermented soybean curd, Sweetened and un-sweetened soy milk, Soy milk powder, Broad-bean paste |
| 15. Nuts and seeds | Sesame, Sunflower, Watermelon seeds, Lotus seeds, Peanuts, Walnuts, Almonds, Hazelnuts, Pine-nuts, Pistachios, Cashew nuts, Peanut butter |
| 16. Fruits and fresh juices | Fresh fruits, Fresh fruit juices |
| 17. Preserved fruit | Dried and canned fruit (added sugar), Dried dates, Dried longan |
| 18. Red meats | Pork, Beef, Lamb, Donkey, Rabbit |
| 19. Poultry | Chicken, Duck, Goose |
| 20. Organ meats | Liver, Kidney, Large intestine, Ear, Feet, Pork blood curd, Duck blood curd, Pork tendon, Beef tendon |
| 21. Processed meats | Sausages, Ham, Luncheon meat, Dried meat, Smoked meat, Salted meat |
| 22. Eggs and its products | Whole eggs, Yolk, White, Preserved eggs |
| 23. Fish and seafood | Fresh- and salt-water fish, Dried fish, Shellfish |
| 24. Low-fat dairy products | Skim or low-fat milk, Yogurt, Cottage cheese, Low-fat cheese |
| 25. High-fat dairy products | Whole milk, Whole milk powder, Cream cheese. Cream, Butter, Ice cream |
| 26. Western-style fast-food | Fried chicken, Sandwich, Hamburger, Hotdog, Pizza |
| 27. Instant foods | Instant noodles, Instant multigrain porridge, Refined grain ready-to-eat cereals, Frozen dumplings |
| 28. Sweets and snacks | Biscuit, Cracker, Jelly, Jam, Chocolate, Honey, Sugar, Candies, Cake, Cookies, Pie, Pastries, Corn Crisps, Onion Rings, Potato Chips, Popcorn |
| 29. Sugar-sweetened beverages | Fruit or flavored drinks, Soft drinks |
| 30. Coffee and tea | Tea, Coffee |
| 31. Alcoholic beverages | Liquors, Wine, Vodka, Cocktails, Whiskey, Beer |
| 32. Condiments and spice | Soy sauce, Vinegar, Salt, Star anise, White pepper, Mayonnaise |
| 33. Vegetable oils | Soybean oil, Tea oil, Rapeseed oil, Olive oil |
| 34. Animal oils | Beef tallow, Duck fat |
| 35. Others | Semen Cassiae, Dried Tangerine, Momordica Grosvenori |
