# Supplementary material for: Associations of dietary patterns with obesity and weight change for adults aged 18–65 years: Evidence from the China Health and Nutrition Survey (CHNS)
Source: PLoS One. 2023 Jan 25;18(1):e0279625. doi: 10.1371/journal.pone.0279625 (PMC9876275; doi:10.1371/journal.pone.0279625)
Supplement: S3 Table — (DOCX) [file pone.0279625.s003.docx]

| **S3 Table. Relative risks of incident obesity according to quintiles of dietary patterns in women** | | | | | | | |
| --- | --- | --- | --- | --- | --- | --- | --- |
|  | **Women (n = 2,771)** | | | | | | |
|  | **Quintile 1** | **Quintile 2** | **Quintile 3** | **Quintile 4** | **Quintile 5** | **P for trend^d^** | **Per one unit increase** |
| **Westernized dietary pattern** | | | | | | | |
| Person-years of follow up | 6483.04 | 5353.2 | 4803.28 | 4054.95 | 2994.54 |  |  |
| Cases. No. | 154 | 160 | 159 | 148 | 106 |  |  |
| Model 1^a^ | 1.00 | 1.34 (1.07-1.67) | 1.53 (1.22-1.91) | 1.72 (1.37-2.16) | 1.73 (1.34-2.23) | <.001 | 1.21 (1.12-1.31) |
| Model 2^b^ | 1.00 | 1.35 (1.08-1.69) | 1.54 (1.23-1.93) | 1.73 (1.37-2.18) | 1.74 (1.34-2.27) | <.001 | 1.22 (1.12-1.32) |
| Model 3^c^ | 1.00 | 1.27 (1.01-1.60) | 1.39 (1.10-1.78) | 1.57 (1.22-2.03) | 1.55 (1.14-2.11) | 0.004 | 1.16 (1.05-1.28) |
| **Traditional Chinese dietary pattern** | | | | | | | |
| Person-years of follow up | 5144.9 | 4758.54 | 4852.7 | 4677.54 | 4255.33 |  |  |
| Cases. No. | 198 | 134 | 132 | 144 | 119 |  |  |
| Model 1^a^ | 1.00 | 0.71 (0.57-0.89) | 0.70 (0.56-0.87) | 0.83 (0.67-1.03) | 0.76 (0.61-0.96) | 0.025 | 0.93 (0.87-1.00) |
| Model 2^b^ | 1.00 | 0.71 (0.57-0.89) | 0.70 (0.56-0.88) | 0.83 (0.67-1.04) | 0.77 (0.61-0.97) | 0.035 | 0.94 (0.87-1.01) |
| Model 3^c^ | 1.00 | 0.72 (0.57-0.92) | 0.77 (0.61-0.99) | 0.92 (0.72-1.16) | 0.82 (0.63-1.05) | 0.233 | 0.95 (0.87-1.03) |
| **High-starch plant-based dietary pattern** | | | | | | | |
| Person-years of follow up | 5603.65 | 4755.9 | 4334.29 | 4214.66 | 4780.51 |  |  |
| Cases. No. | 160 | 144 | 127 | 127 | 169 |  |  |
| Model 1^a^ | 1.00 | 1.07 (0.85-1.34) | 1.03 (0.81-1.30) | 1.02 (0.81-1.30) | 1.14 (0.92-1.42) | 0.284 | 1.01 (0.94-1.08) |
| Model 2^b^ | 1.00 | 1.07 (0.85-1.34) | 1.03 (0.81-1.30) | 1.03 (0.81-1.31) | 1.15 (0.93-1.43) | 0.254 | 1.01 (0.94-1.08) |
| Model 3^c^ | 1.00 | 1.02 (0.81-1.29) | 0.95 (0.74-1.21) | 0.95 (0.74-1.21) | 1.05 (0.83-1.32) | 0.792 | 0.98 (0.91-1.05) |
| Abbreviations: RR, relative risk; CI, confidence interval.  ^a^Model 1 was adjusted for age (continuous, years), and energy intake (continuous, kcal/d).  ^b^Model 2 was adjusted for age (continuous, years), energy intake (continuous, kcal/d), physical activity (0, 0 <- 18, 18 <- 36, and > 36 MET-h/week), smoking status (0, 0 <- 10, 10 <- 20, 20 <- 30, and > 30 pack-years), and alcohol drinking (0, 0 <- 6, 6 <- 12, 12 <- 24, and > 24 g/d).  ^c^Model 3 was adjusted for age (continuous, years), energy intake (continuous, kcal/d), physical activity (0, 0 <- 18, 18 <- 36, and > 36 MET-h/week), smoking status (0, 0 <- 10, 10 <- 20, 20 <- 30, and > 30 pack-years), alcohol drinking (0, 0 <- 6, 6 <- 12, 12 <- 24, and > 24 g/d), region (Northern region, Eastern region, Western region, and Central region), education level (illiteracy, primary school, junior high school, and high school or higher), marital status (never married, married, and divorced), household income per capita inflated to 2015 (tertile, RMB), and urbanization index (tertile).  ^d^P for trend was calculated using the median value of each quintile category as a continuous variable. | | | | | | | |
