# Supplementary material for: Associations of dietary patterns with obesity and weight change for adults aged 18–65 years: Evidence from the China Health and Nutrition Survey (CHNS)
Source: PLoS One. 2023 Jan 25;18(1):e0279625. doi: 10.1371/journal.pone.0279625 (PMC9876275; doi:10.1371/journal.pone.0279625)
Supplement: S8 Table — (DOCX) [file pone.0279625.s008.docx]

| **S8 Table. Least-squares means of body weight change (kg/5 years) among participants who reported baseline BMI < 30 kg/m^2^** | | | | | | |
| --- | --- | --- | --- | --- | --- | --- |
|  | **Total population (n = 6,461)** | | | | | |
|  | **Quintile 1** | **Quintile 2** | **Quintile 3** | **Quintile 4** | **Quintile 5** | **P for trend^d^** |
| **Westernized dietary pattern** | | | | | | |
| Weight change, median (kg/5 years) | 1.07 | 1.12 | 1.00 | 1.11 | 0.99 |  |
| Model 1^a^ | 1.47 (1.09-1.84) | 1.23 (0.86-1.61) | 1.28 (0.91-1.66) | 1.64 (1.27-2.02) | 1.33 (0.96-1.70) | 0.926 |
| Model 2^b^ | 2.07 (1.37-2.76) | 1.81 (1.15-2.48) | 1.88 (1.21-2.54) | 2.24 (1.56-2.91) | 1.80 (1.11-2.50) | 0.734 |
| Model 3^c^ | 1.62 (0.93-2.31) | 1.50 (0.84-2.16) | 1.73 (1.07-2.39) | 2.13 (1.46-2.80) | 1.79 (1.11-2.48) | 0.353 |
| **Traditional Chinese dietary pattern** | | | | | | |
| Weight change, median (kg/5 years) | 1.14 | 1.08 | 0.99 | 1.00 | 1.04 |  |
| Model 1^a^ | 1.31 (0.94-1.69) | 1.62 (1.24-1.99) | 1.38 (1.00-1.75) | 1.37 (1.00-1.74) | 1.28 (0.91-1.66) | 0.719 |
| Model 2^b^ | 1.90 (1.22-2.58) | 2.13 (1.46-2.81) | 1.92 (1.26-2.58) | 1.95 (1.28-2.62) | 1.86 (1.19-2.53) | 0.771 |
| Model 3^c^ | 1.86 (1.19-2.54) | 1.97 (1.31-2.64) | 1.62 (0.96-2.28) | 1.63 (0.97-2.30) | 1.65 (0.99-2.31) | 0.285 |
| **High-starch plant-based dietary pattern** | | | | | | |
| Weight change, median (kg/5 years) | 0.91 | 0.98 | 1.21 | 1.14 | 1.10 |  |
| Model 1^a^ | 1.12 (0.74-1.50) | 1.15 (0.78-1.52) | 1.43 (1.06-1.81) | 1.73 (1.36-2.11) | 1.52 (1.14-1.89) | 0.039 |
| Model 2^b^ | 1.72 (1.05-2.38) | 1.77 (1.10-2.44) | 1.99 (1.33-2.66) | 2.28 (1.62-2.94) | 1.97 (1.30-2.64) | 0.174 |
| Model 3^c^ | 1.57 (0.91-2.24) | 1.56 (0.90-2.23) | 1.78 (1.12-2.44) | 2.01 (1.36-2.67) | 1.77 (1.10-2.43) | 0.265 |
| Abbreviations: LS-mean, least-squares mean; CI, confidence interval.  ^a^Model 1 was adjusted for sex (men, women), age (continuous, years), and energy intake (continuous, kcal/d).  ^b^Model 2 was adjusted for sex (men, women), age (continuous, years), energy intake (continuous, kcal/d), physical activity (0, 0 <- 18, 18 <- 36, and > 36MET-h/wk), smoking status (0, 0 <- 10, 10 <- 20, 20 <- 30, and > 30 pack-years), alcohol drinking(0, 0 <- 6, 6 <- 12, 12 <- 24, and > 24 g/d), region (Northern region, Eastern region, Western region, and Central region), education level (illiteracy, primary school, junior high school, and high school or higher), marital status (never married, married, and divorced), household income per capita inflated to 2015 (tertile, RMB), and urbanization index (tertile).  ^c^Model 3 was adjusted for baseline body weight (continuous, kg) in addition to variables in Model 2.  ^d^P for trend was calculated using the median value of each quintile category as a continuous variable. | | | | | | |
